# Born to sweet delight: Using natural models of malaria protection to understand and neutralize *P*. *falciparum* pathogenesis

**DOI:** 10.1371/journal.ppat.1007770

**Published:** 2019-06-20

**Authors:** Joseph W. Saelens, Steve M. Taylor

**Affiliations:** 1 Division of Infectious Diseases, Duke University School of Medicine, Durham, North Carolina, United States of America; 2 Duke Global Health Institute, Duke University, Durham, North Carolina, United States of America; University of Wisconsin Medical School, UNITED STATES

## Introduction

Across millennia of coevolution, malaria parasites have mediated their human hosts’ patterns of settlement, authored their historical milestones, and shaped their genome. The human genome harbors archived responses to the pressure of severe, life-threatening malaria, and chief among these are innate variants of erythrocytes, which serve as principal cellular hosts for the parasite. Since JBS Haldane first speculated that red blood cell (RBC) variants may confer some resistance to malaria, numerous studies using diverse approaches have identified an array of innate variants, which can be grouped as disorders of the erythrocyte cytoskeleton (e.g., Southeast Asian ovalocytosis), variation in erythrocyte surface antigens (e.g., ABO and Duffy), enzymatic aberrations (e.g., glucose-6-phosphate dehydrogenase deficiency), and mutants of α- or β-globin proteins, either as deletions (e.g., α-thalassemia) or point mutations (e.g., hemoglobin [Hb] S or C). To varying degrees, each of these variants of erythrocytes have been selected over generations owing to enhanced fitness when human populations are exposed to intense malaria transmission.

These variants can provide striking protection: as one example, in African children, heterozygosity for sickle Hb reduces the risk of severe, life-threatening malaria by over 90% [1]. This degree of protection against severe malaria exceeds any other preventive medical intervention, including the 34% protection conferred by the most advanced malaria vaccine, RTS,S/AS01 [2]. Because of this, these models of naturally occurring protection need to be exploited to better understand the mechanisms of parasite pathogenesis and how these can be neutralized. Now, nearly 7 decades following Haldane’s hypothesis, there exists a pressing need to translate these observations into strategies to interrupt malaria and render fewer children born to the endless night of immiserating malaria.

## Historical perspectives

Malaria is transmitted between humans by *Anopheles* spp. mosquitos and is caused by five species of *Plasmodium* spp. parasites—*Plasmodium falciparum*, *P*. *vivax*, *P*. *ovale*, *P*. *malariae*, and *P*. *knowlesi*. *P*. *falciparum* and *P*. *vivax* are distributed most widely throughout the tropics and cause most human disease. *P*. *vivax* is very rare in most of Africa because most Africans lack expression on their erythrocytes of the Duffy antigen receptor for chemokines (DARC), which enables the efficient invasion of red cells by *P*. *vivax*. Globally, *P*. *falciparum* is the most common and the deadliest species, and it kills over 400,000 people annually, mostly children in sub-Saharan Africa.

After a short stage in hepatocytes, the principal target in humans is the erythrocyte, which serves as the primary platform for parasite propagation. Consequently, variants of erythrocytes are the principal known means by which humans have developed protection, most notably through mutations in Hb (**Table 1**). Hb is formed by a tetramer of two α- and two β-globin proteins; mutations of Hb result from either deletion of globin gene copies (in the thalassemias) or in coding point mutations in β-globin. A wide variety of point mutations in β-globin have been described, and most important for malaria protection are the substitutions of glutamate at position 6 for either valine (to produce HbS) or lysine (HbC). The inheritance of two mutated β-globins (as either HbSS or HbSC) produces sickle cell anemia and significant multisystem morbidity; in contrast, the inheritance of either sickle-cell trait (HbAS) or homozygous HbC (HbCC) produces little morbidity but substantial protection.

**Table 1:** Common Hb variants and their phenotypes.

Variant	Epidemiology	Genotype	Clinical phenotype
α-thalassemias			
α^+^-thal trait	Global	Loss of one α-globin gene (αα/α-)	Asymptomatic
α^0^-thal trait	Global	Loss of two α-globin genes (αα/—)	Mild anemia
HbS	West, central, and East Africa; Arabian Peninsula; Southeast Asia	Glu → Val at position 6 of β-globin	HbSS: sickle cell disease with pain crises, transfusions, acute chest syndrome, stroke HbAS: largely asymptomatic
HbC	West African Sahel	Glu → Lys at position 6 of β-globin	HbCC: mild hemolysis and anemia HbAC: asymptomatic
HbE	Southeast Asia	Glu → Lys at position 26 of β-globin	HbEE: minimal anemia HbAE: asymptomatic
HbF	Produced in first 6 months of life	Normal	Greater oxygen affinity than adult HbA

Abbreviations: Glu, glutamate; Hb, hemoglobin; Lys, lysine; Val, valine.

## Clinical epidemiology

Interestingly, both HbAS and HbCC specifically protect children from severe disease, suggesting that they attenuate the specific pathogenic mechanisms of either the parasite or host while allowing children to be infected with *P*. *falciparum*. This conclusion is supported by contrasts between the degrees of protection conferred by HbAS against severe malaria (91%), uncomplicated malaria (31%), and asymptomatic parasitization (none) [1]. The risk of severe malaria is also reduced by homozygous (37%) or heterozygous (17%) α-thalassemia, but these variants confer no protection from uncomplicated malaria. In addition to their individual effects, epistasis has been repeatedly reported between HbAS and α-thalassemia, in which the coinheritance of both mutations negates the protective effects of either against severe malaria [3]. This clear biological and clinical interaction between the two variants suggests that candidate molecular mechanisms of protection should follow a similar pattern.

## In vitro effects

Numerous in vitro studies have reported the effects of Hb variants on parasite cellular phenotypes. Generally, in vitro studies have rejected the hypothesis that parasites are unable to invade hemoglobinopathic RBCs: invasion has been reported as normal for HbAS [4], HbCC [5], HbAC [5], HbAE, and HbEE cells [6], as well as for α-thalassemic RBCs [7] and those containing HbF [8]. Studies of parasite growth have been conflicting: early studies [4, 9, 10] reported that, compared with HbAA RBCs, growth of parasites was attenuated specifically at low oxygen tension (approximately 5%) in HbAS or HbCC RBCs, but more recent studies have reported equivalent growth in HbAA, HbAS, and HbAC RBCs [11, 12] in similarly hypoxic environments. However, a recent, more detailed investigation of the stage-specific growth effects of hypoxia in HbAS cells reported that hypoxia attenuates parasite maturation specifically in the latter stages of parasite development, during which infected erythrocytes typically sequester in hypoxic deep vascular beds [13]. How this phenomenon may operate in vivo or be present in other Hb variants remain open questions.

## Cellular pathogenesis

Cellular mechanisms of pathogenesis are principally governed by interactions between infected RBCs (iRBCs) and extracellular ligands on host cells, including endothelium, leukocytes, and uninfected RBCs; these interactions are enabled by the expression of parasite-derived proteins on the RBC surface, including the hypervariable protein families *P*. *falciparum* erythrocyte membrane protein 1 (PfEMP1), repetitive interspersed families of polypeptides (RIFIN), and subtelomeric variable open reading frame (STEVOR). This cytoadherence allows iRBCs to sequester in the microvasculature and avoid splenic clearance, attenuate immune activation [14], and activate endothelial cells [15]. In vitro studies reveal that HbAS and HbCC reduce the cytoadherence of iRBCs to human microvascular endothelial cells and produce aberrant expression of PfEMP1 on the iRBC surface compared with HbAA [16, 17]. The reduction in PfEMP1 expression is important because PfEMP1 variants enable the interaction of iRBCs with endothelial receptors including endothelial protein C receptor (ePCR), intercellular adhesion molecule 1 (ICAM-1), and CD36 that are increasingly recognized as important in the pathogenesis of severe malaria [18]. Intriguingly, a more recent study reported that whereas HbAS reduced PfEMP1 expression, adherence to recombinant ICAM-1 and CD36, and adherence to uninfected RBCs, these phenotypic effects were reversed by coinheritance with α-thalassemia [19], mirroring the clinical epidemiology of HbAS, α-thalassemia, and malaria risk [3]. These attenuated interactions have functional consequences: in a recent study, HbAS reduced the ability of iRBCs to activate endothelium, possibly as a result of the reduced quality of adhesion of the iRBC to endothelium [20]. Taken together, these data suggest that the ability of HbCC and HbAS to disrupt *P*. *falciparum’s* ability to interact with extracellular ligands contributes significantly to their protection from severe malaria.

## Molecular pathogenesis

As noted above, *P*. *falciparum* exports a large number of proteins to the iRBC surface, and a principal component of the export machinery is Maurer’s clefts, the parasite-derived Golgi-like membranous structures that serve as transport hubs for PfEMP1 and other exported proteins. As the parasite matures in the RBC, these Maurer’s clefts are conveyed along a scaffold of host-cell actin that the parasite has repurposed to assemble a transport network; in HbCC and HbSC RBCs, Maurer’s clefts are dysmorphic, the actin assemblage is disorganized [21], Maurer’s cleft motion is impaired [22], and protein export to the RBC surface is delayed [23]. Some of these phenotypes have been induced by the exposure of HbAA RBCs to oxidation prior to infection, suggesting that oxidative insult from β-globin variants is an important contributor [11]. In a separate line of inquiry, host microRNAs (miRNAs) harbored within HbAS RBCs attenuated parasite growth and perturbed parasite protein translation owing to the production of chimeric mRNAs formed by the fusion of host miRNA and parasite mRNA transcripts [24], suggesting a novel mechanism of interactions between host RBC and parasite. Collectively, these studies suggest that HbAS exerts pervasive effects on parasite mechanisms and underscores the efficiency of this system for exploring fundamental mechanisms of parasite pathogenesis (**Box 1**).

**Box 1. Unanswered questions for protection**

1. How does reduced PfEMP1 expression and cytoadherence modify the effects of hypoxia-induced arrest of maturation in HbAS RBCs?
2. Do host miRNAs influence the quality or quantity of surface antigen expression or cytoadherence in HbAS RBCs?
3. How do β-globin variants disrupt the assembly of the actin cytoskeleton and Maurer’s clefts during ring-stage development?
4. Do β-globin variants attenuate the interaction of infected RBCs with host immune cells?

## A pathway to progress

Just as pathogen drug-resistance phenotypes furnish a model by which to better understand drug mechanisms of action, human disease resistance enables us to more efficiently explore pathogen mechanisms of disease and thereby identify targets for intervention. There are two models for this approach. The first model relates to *P*. *vivax* malaria. As noted above, many Africans are protected from *P*. *vivax* because the absence of DARC on the erythrocyte surface prevents RBC invasion by *P*. *vivax*. This model of protection enabled the identification of the parasite-derived binding partner for DARC: *P*. *vivax* Duffy binding protein (PvDBP). In populations exposed to *P*. *vivax*, naturally occurring antibodies to PvDBP are associated with protection from vivax malaria (in DARC-positive people), and this apparent functional protection has prioritized PvDBP as an advanced candidate antigen for vivax-specific vaccination strategies [25]. The second model relates to pregnancy-associated *P*. *falciparum* malaria. Epidemiologically, pregnant women are at high risk in endemic areas for falciparum malaria but develop over successive pregnancies acquired immunity to its consequences, including placental malaria. Placental malaria results from interactions between chondroitin sulfate A in the syncytiotrophoblast and a parasite-derived surface protein VAR2CSA; this essential interaction, and the model of acquired immunity to it, enabled the identification of functional antibody responses that attenuate placental malaria. Currently, two early-phase vaccines are being developed to generate responses directed against VAR2CSA and protect women and their offspring from placental malaria.

Adaptive evolution in human populations has furnished to us natural strategies by which to neutralize malaria parasites in the form of erythrocyte variants. By using the protective phenotypes to infer essential mechanisms of disease, we can rationally develop new approaches to preventing and treating this ancient and immiserating disease.
